# Bromine Incorporation Affects Phase Transformations and Thermal Stability of Lead Halide Perovskites

**DOI:** 10.1021/jacs.4c04508

**Published:** 2024-06-27

**Authors:** Diana K. LaFollette, Juanita Hidalgo, Omar Allam, Jonghee Yang, Austin Shoemaker, Ruipeng Li, Barry Lai, Benjamin Lawrie, Sergei Kalinin, Carlo A. R. Perini, Mahshid Ahmadi, Seung Soon Jang, Juan-Pablo Correa-Baena

**Affiliations:** † School of Materials Science and Engineering, Georgia Institute of Technology, North Ave NW, Atlanta, Georgia 30332, United States; ‡ Woodruff School of Mechanical Engineering, Georgia Institute of Technology, North Ave NW, Atlanta, Georgia 30332, United States; § Department of Chemistry, Yonsei University, Seoul 03722, Republic of Korea; ∥ Institute for Advanced Materials and Manufacturing Department of Materials Science and Engineering, University of Tennessee, Knoxville, Knoxville, Tennessee 37996, United States; ⊥ National Synchrotron Light Source II, Brookhaven National Lab, Upton, New York 11973, United States; # Advanced Photon Source, Argonne National Laboratory, Lemont, Illinois 60439, United States; % Center for Nanophase Materials Sciences, Oak Ridge National Laboratory, Oak Ridge, Tennessee 37831, United States; & Materials Science and Technology Division, Oak Ridge National Laboratory, Oak Ridge, Tennessee 37831, United States; $ Department of Materials Science and Engineering, University of Tennessee, Knoxville, Tennessee 37996 United States; @ Physical Sciences Division, Pacific Northwest National Laboratory, Richland, Washington 99352 United States; ! School of Chemistry and Biochemistry, Georgia Institute of Technology, North Ave NW, Atlanta, Georgia 30332, United States

## Abstract

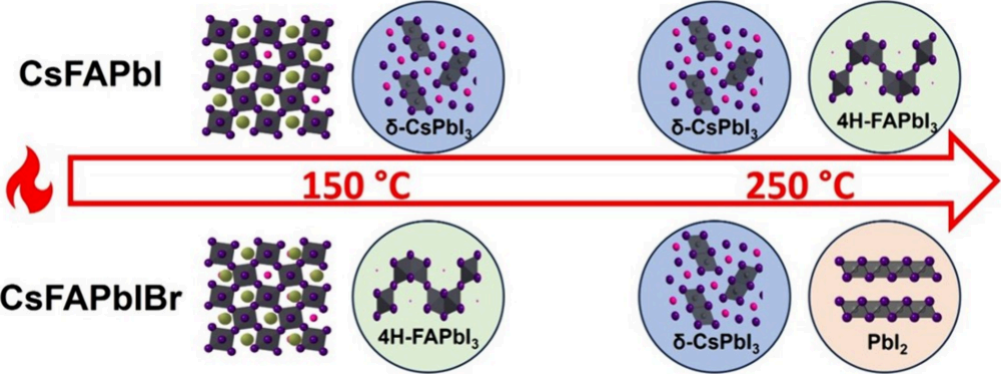

Mixed-cation and mixed-halide lead halide perovskites show great potential for their application in photovoltaics. Many of the high-performance compositions are made of cesium, formamidinium, lead, iodine, and bromine. However, incorporating bromine in iodine-rich compositions and its effects on the thermal stability of the perovskite structure has not been thoroughly studied. In this work, we study how replacing iodine with bromine in the state-of-the-art Cs_0.17_FA_0.83_PbI_3_ perovskite composition leads to different dynamics in the phase transformations as a function of temperature. Through a combination of structural characterization, cathodoluminescence mapping, X-ray photoelectron spectroscopy, and first-principles calculations, we reveal that the incorporation of bromine reduces the thermodynamic phase stability of the films and shifts the products of phase transformations. Our results suggest that bromine-driven vacancy formation during high temperature exposure leads to irreversible transformations into PbI_2_, whereas materials with only iodine go through transformations into hexagonal polytypes, such as the 4H-FAPbI_3_ phase. This work sheds light on the structural impacts of adding bromine on thermodynamic phase stability and provides new insights into the importance of understanding the complexity of phase transformations and secondary phases in mixed-cation and mixed-halide systems.

## Introduction

Perovskite solar cells (PSCs) are a promising photovoltaic technology due to their high efficiencies and low cost, but they lack long-term stability compared to commercially available silicon solar cells.^1,2^ This is partially due to the thermodynamic phase instability of the corner-sharing crystal structure at room temperature.^2−8^ Many studies have presented the temperature phase diagrams of single-cation mixed-halide lead halide perovskites (LHPs) and mixed-cation and single-halide LHPs.^3,9−12^ However, some of the highest performing compositions for efficiency and stability are both mixed-cation and mixed-halide, such as Cs_0.17_FA_0.83_PbI_3_ (CsFAPbI) and Cs_0.17_FA_0.83_Pb(I_0.83_Br_0.17_)_3_ (CsFAPbIBr).^13^ Unraveling understanding of the mechanisms underlying thermodynamic phase stability and phase transformations in complex but high performing mixed-cation and mixed-halide systems is essential to ultimately engineer a high performing and stable PSC. Many studies show the benefit of adding dilute amounts of bromine,^14−21^ but it is still not well understood how this addition affects the phase transformations and thermodynamic phase stability in high-performing mixed-cation and mixed-halide systems.

LHPs are compounds with the chemical formula ABX_3_, where the A-site cation is commonly formamidinium (FA^+^), and/or cesium (Cs^+^); the B-site cation is lead (Pb^2+^); and the X-site anion is iodine (I^–^) and/or bromine (Br^–^).^1,2^ LHPs are characterized by corner-sharing PbX_6_ octahedra and some degree of defect tolerance that lead to long-lived charge carriers.^22−24^ However, these corner-sharing octahedra are not always thermodynamically phase stable at room temperature.^22−24^ Inorganic CsPbI_3_ goes through an undesirable phase transformation from the cubic (α) phase to the orthorhombic *Pnma* (δ) phase below 325 °C.^25^ FAPbI_3_ spontaneously transforms from the cubic (α) phase to the undesirable hexagonal phases (2H-, 4H-, and 6H-FAPbI_3_)^26,27^ below 150 °C.^28^ Thermal instability is currently remediated by mixing both Cs and FA cations,^24^ with single crystals of Cs_0.1_FA_0.9_PbI_3_ showing a stable cubic phase at as low as 27 °C.^29^ Single-cation systems have also seen improvement in thermal stability by mixing halides, such as stabilizing the tetragonal (β) phase as low as 100 °C by replacing 17% of iodine with bromine in CsPbI_3._^25^

Size mismatch between cations and/or anions in the ABX_3_ structure causes distortions to the cubic lattice, referred to here as lattice distortion.^30^ Improvements in room temperature phase stability due to mixing cations and/or anions are thought to be due to reducing lattice distortion in the perovskite by offsetting size mismatch.^13,22,24^ Adding bromine to CsFAPbI reduces lattice distortion due to its smaller size compared to iodine.^9,20,21^ By reducing overall lattice distortion, adding bromine to CsPbI_3_ limits phase transformation into orthorhombic edge-sharing non-perovskite phases.^31^ However, introducing a small amount of bromine into an iodine rich perovskite creates areas with local strain in the lattice due to differences in Pb–I–Pb and Pb–Br–Pb bonds.^22,32^ These areas of local strain decrease the activation energy of halide vacancy formation.^32^ Halide vacancies are the initiating step for phase segregation^33^ and degradation into PbI_2_.^34^ The effects of changing composition on the phase stability of the material are a balance between reducing lattice distortion and minimizing local strain that can accelerate degradation via vacancies.^32^ What is left to still be understood is how changing composition in mixed-cation and mixed-halide systems affects temperature-dependent phase transformations.

Stabilizing the LHP corner-sharing octahedra at room temperature is a balance between the thermodynamics of the crystal structure and the integrity of the organic cation. It is not just the thermodynamic phase stability of the crystal structure itself that changes with composition, but the thermal stability of the organic material within the LHP also changes.^35^ There are many studies that focus on the exact decomposition products of the organic cation at different temperatures.^7,36−41^ In pure FA LHPs, the activation energy for mass loss due to thermal decomposition of the organic cation is 115 ± 3 kJ mol^–1^ for FAPbI_3_ and 133 ± 3 kJ mol^–1^ for FAPbBr_3_, indicating greater thermal stability with bromine.^38^ However, similar analysis of the organic precursors shows that the activation energy for degradation of FABr (52 ± 1 kJ mol^–1^) is lower than FAI (77 ± 1 kJ mol^–1^)^38^ and thus degradation of FABr will occur at lower temperature than FAI.^38^ These differences in activation energy for degradation with composition and chemical state will likely also influence the thermal properties of mixed-cation and mixed-halide compositions. In CsFAPbI, there is no measured mass loss as FAI below 200 °C.^35^ However, in the mixed-cation and mixed-halide system CsFAPbIBr the organic is lost at as low as 100 °C in the form of FABr^18,35^ and then beginning at 160 °C in the form of FAI.^35^ LHPs for PSC applications are commonly annealed between 100 and 150 °C,^13,42,43^ indicating that adding bromine could cause loss of organic within this range. This low temperature loss of organic creates vacancies and changes the composition of the thin film, which will affect the final crystal structures in the thin films.^18,35^ The optimal annealing temperature to obtain phase pure LHPs is dependent on the composition.^44^ To ultimately create a stable and efficient solar cell, we must understand how the temperature, composition, and phase transformations are interrelated.

In this work, we study the effects of the incorporation of bromine into an iodine-based perovskite (i.e., CsFAPbI) on temperature-induced phase transformations and degradation. We show that replacing 17% of iodine with bromine shifts the formation of non-perovskite phases from primarily δ-CsPbI_3_ and 4H-FAPbI_3_ in CsFAPbI to primarily PbI_2_ in CsFAPbIBr after high temperature annealing of thin films. X-ray diffraction (XRD) is used to characterize the crystal structure and polycrystalline thin film orientation. The structural information is then correlated to optical and morphological mapping using cathodoluminescence scanning electron microscopy (CL-SEM) and compositional analysis via X-ray photoelectron spectroscopy (XPS). The phase and compositional changes observed with these techniques are then explained with the help of density functional theory (DFT) calculations. Using these techniques, we explain the role of bromine and annealing temperature on phase transformations in mixed-cation and mixed-halide perovskites and how they impact solar cell performance. We reveal that adding 17% molar bromine causes loss of halides in CsFAPbIBr at as low as 100 °C while the loss of halides in CsFAPbI begins above 150 °C. The focus of this paper is not on how the halides are lost or their exact chemical state but the effect of the resultant vacancies. For this work, simultaneous loss of organics and halides will be termed FAI or FABr. The increased production of halide vacancies promotes the formation of secondary phases at lower temperatures in CsFAPbIBr compositions, offsetting the potential benefits of minimizing lattice distortion and reducing solar cell performance.

## Experimental Section

### Thin Film Processing

Stoichiometric 1.2 M Cs_0.17_FA_0.83_PbI_3_ (CsFAPbI) and Cs_0.17_FA_0.83_Pb(I_0.83_Br_0.17_)_3_ (CsFAPbIBr) precursor solutions were made by mixing precursor powders and solvents inside a nitrogen glovebox_._ Solutions were made by combining FAI (Sigma-Aldrich), CsI (Sigma-Aldrich), PbI_2_ (Sigma-Aldrich), and PbBr_2_ (Sigma-Aldrich) powders in appropriate molar ratios. Powders were dissolved in 2:1 v/v dimethylformamide (DMF) and dimethyl sulfoxide (DMSO). Films were then spin-coated on 1 in.^2^ soda lime glass in a nitrogen glovebox at 1000 rpm for 10 s, followed by 6000 rpm for 20 s and adding 250 μL of chlorobenzene (Sigma-Aldrich) 5 s before the end of the spin-coating process. Perovskite thin films were annealed at 65, 100, 150, 200, or 250 °C for 10 min inside the nitrogen glovebox. The films were characterized after cooldown at the end of the annealing step (ex situ), as LHPs are known to change their crystal structure during cooling.^45^ This work focuses on the crystal phases present in a thin film that would then be used in perovskite solar cells.^43^ Thin films are naturally more prone to defects and degradation than powder or single crystals that undergo the same temperature transitions.

### Solar Cells Fabrication and Characterization

Full devices were made in the fluorinated tin oxide coated glass/compact-TiO_2_/mesoporous-TiO_2_/perovskite/2,2′,7,7′-tetrakis[*N*,*N*-di(4-methoxyphenyl)amino]-9,9′-spirobifluorene/Au n–i–p architecture.^43^ Device performance was tested using a Litos Lite (Fluxim, Switzerland) with a Wavelabs Sinus-70 AAA solar simulator with an illumination of AM 1.5 G at room temperature in ambient air. The current density–voltage (*J–V)* curves were measured from 1.4 to −0.5 V with a scan speed of 10 mV s^–1^. The active area of the device was 0.128 cm^2^ with a mask of 0.0625 cm^2^. Intensity-modulated photocurrent spectroscopy (IMPS) was carried out using Paios hardware (Fluxim, Switzerland) on full devices under 1 sun illumination in ambient air and were measured from 1 Hz to 1 MHz using 50 points.

### Morphological Measurements and Correlation to Local Performance

CL-SEM measurements were carried out at the Center for Nanophase Materials Sciences at Oakridge National Laboratory. CsFAPbI/CsFAPbIBr thin films were fabricated on unpatterned FTO. An FEI Quattro environmental SEM with a Delmic Sparc CL collection module was used with a parabolic mirror to collect the CL signals from the film after excitation. An electron beam with an acceleration voltage of 5 kV and a beam current of 32 pA was passed through a hole in the parabolic mirror for sample excitation. The CL signal collection acquisition time was 400 ms per spectrum with a pixel size of 40 nm. All measurements were conducted in a low-vacuum environment of 50 Pa H_2_O vapor. The combination of this energy, current, and vacuum was chosen to mitigate sample charging and damage while still collecting measurable intensities.^46,47^ Data was then processed on google Colab using Python 3.6 and the scikit-learn 0.22.1 library.

### Structural Characterization

XRD measurements were performed at Georgia Tech in the Institute for Electronics and Nanotechnology facilities. Measurements were taken under ambient conditions on the Malvern PANalytical Empyrean with Bragg–Brentano geometry using a Cu Kα source. CsFAPbI/CsFAPbIBr films were fabricated on soda lime glass.

### Density Functional Theory Methods

All density functional theory (DFT) calculations were performed using Vienna Ab initio Simulation Package (VASP).^48^ The exchange-correlation interaction was described using the generalized gradient approximation (GGA) Perdew–Burke–Ernzerhof (PBE) functional^49−51^ with the projector augmented wave (PAW) method.^52^ Our objective of our study was to understand the impact of bromine insertion, utilizing a computationally accessible model, CsFA_3_Pb_4_I_12_, rather than precisely replicating experimental stoichiometries. Namely, the purpose of our calculations is to elucidate how the inclusion of bromine generally affects vacancy formation and correspondingly how that facilitates phase transformations. XRD patterns of these compositions, showing their similarity in phase transformation pathways, are found in Figures S1 and S2. We initiated with the pure iodine structure. Bromine was successively introduced at different lattice sites, and the configuration yielding the lowest energy was chosen as the most favorable insertion site. This procedure was iterated for multiple bromine insertions. A similar approach was applied to introduce vacancies, where various lattice sites were assessed, and the minimum-energy configurations were selected. All structures were optimized using various k-point space samplings to maintain a consistent grid density of approximately 0.03 Å^–1^ in reciprocal space: 4 × 4 × 3, 4 × 4 × 2, 7 × 3 × 2, and 9 × 9 × 5 for the β-perovskite, 4H-FAPbI_3_, δ-CsPbI_3_, and PbI_2_ phases, respectively. The plane-wave basis set was employed with an energy cutoff of 400 eV. For cell optimization calculations, the energy cutoff was set at 520 eV. The convergence criterion for the electronic self-consistent field (SCF) calculations was fixed at 10^–5^ eV, while the force criterion for ionic relaxation was set to 0.01 eV/Å. To simulate the perovskite surface, we constructed a slab model from the optimized bulk structure. This slab incorporated four lead atomic planes, resulting in a thickness of over 20 Å, which ensured that a representative surface captures multiple repeat units of the perovskite. The slabs had a vacuum gap of over 25 Å to minimize the interfering interaction between periodic images and thereby obtain accurate surface energetics.

### Chemical Information and Correlation to Performance

XPS measurements were conducted utilizing a Thermo Scientific K-Alpha X-ray photoelectron spectrometer. For survey scans, two measurements were averaged with a pass energy of 200 eV and an energy step size of 1 eV. Elemental scans employed a pass energy of 50 eV and an energy step size of 0.1 eV. Specifically, 10 scans were employed for Br, 5 for Pb, 15 for C, 10 for N, 15 for O, 4 for I, and 10 for Cs. The Pb-X peak position served as the reference for binding energies across different samples due to the presence of organics in the films, rendering the use of the C–C peak position from contaminants unreliable. Elemental composition analysis was conducted by using the Thermo Scientific Avantage data system for surface analysis.

X-ray fluorescence (XRF) and X-ray beam induced current (XBIC) mapping measurements were carried out at the Advanced Photon Source at Argonne National Laboratory at beamline 2-ID-D. CsFAPbI/CsFAPbIBr films for XRF were fabricated on glass, and XBIC measurements were done on full devices. Synchrotron X-ray energy was 14 keV with a 0.15 μm step size and a 50 ms dwell time. For XBIC, the measurement was performed by positioning the Au contact facing the incident beam with the current and fluorescence simultaneously collected point by point during mapping with arbitrary intensity units. This setup allows for a direct correlation between XRF and XBIC. The MAPS software was used for data analysis and spectrum fitting to deconvolute overlapping peaks and background from fluorescence data. In addition, after a standard calibration, it was possible to use the software to quantify the mass concentration in the sample and accurately calculate the ratio between the elements. The NIST thin-film standards SRM 1832 and 1833 were used for calibration of the elemental concentrations.

## Results and Discussion

**Figure 1:**
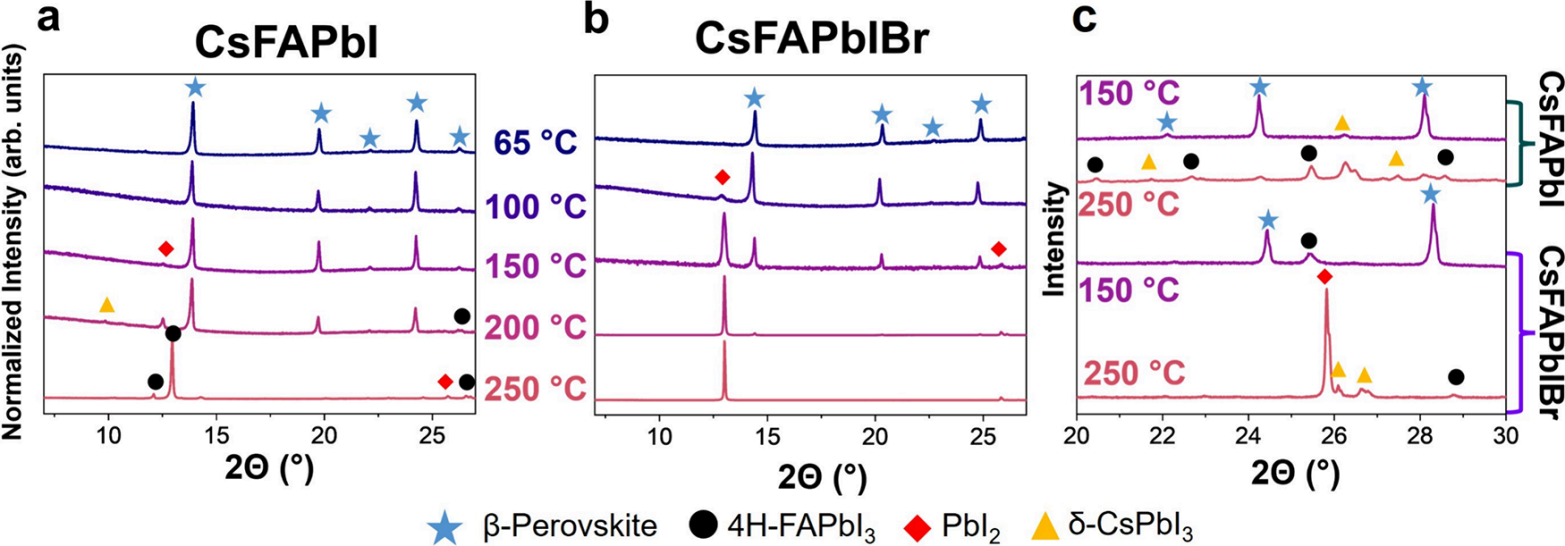
Structural changes as a function of temperature. (a, b) XRD patterns of Cs_0.17_FA_0.83_PbI_3_ (CsFAPbI) and Cs_0.17_FA_0.83_Pb(I_0.83_Br_0.17_)_3_ (CsFAPbIBr) as a function of ex-situ annealing temperature. (c) Zoom-in on high angles of XRD patterns of CsFAPbI and CsFAPbIBr at 150 and 250 °C.

To understand the phase transformations and thermal degradation of CsFAPbI and CsFAPbIBr thin films, we carried out XRD of both compositions over five annealing temperatures (Figure 1a,b). These temperatures range from the lowest commonly used annealing temperature (65 °C)^13^ to 250 °C, which is below the temperature where major gas decomposition products of pure FAPbI_3_ are observed.^53^ For CsFAPbI (Figure 1a), the β-perovskite phase makes up the majority of the diffraction signal below 250 °C, as shown by the characteristic diffraction peaks (2Θ ≃ 14°, 20°, 22°, 24°, and 26°,^27^ blue star in Figure 1). Secondary phases begin appearing at 150 °C with a weak signal from PbI_2_ (2Θ ≃ 12.9°,^54^ red diamond in Figure 1). At 200 °C, CsFAPbI begins to form both δ-CsPbI_3_ and 4H-FAPbI_3_. δ-CsPbI_3_ can be identified by the characteristic peak at 2Θ ≃ 10°^29^ (yellow triangle in Figure 1). The 4H-FAPbI_3_ can be identified by the characteristic peaks at 2Θ ≃ 13° and 26°^55^ (black circle in Figure 1). Overall, as the annealing temperature increases, CsFAPbI forms secondary phases, transforming to δ-CsPbI_3_ and 4H-FAPbI_3_ by 250 °C.

In contrast, the addition of bromine (CsFAPbIBr) leads to a shift in the phase transformations, where the perovskite phase undergoes a phase transformation into PbI_2_ at as low as 100 °C. These secondary phases do not undergo the transition from PbI_2_ to 4H-FAPbI_3_ at higher temperatures as seen in CsFAPbI. The characteristic peaks between 2Θ ≃ 11°–13° are hard to differentiate between 4H-FAPbI_3_ and PbI_2_ in isolation. However, the two phases can be distinguished by looking at the higher angle diffraction peaks (Figure 1c). Diffraction at these higher angles in both CsFAPbI and CsFAPbIBr is dominated by the β-perovskite phase at 150 °C, with some influence of secondary phases. However, at 250 °C, CsFAPbI has many lower intensity peaks that can be attributed to δ-CsPbI_3_ and 4H-FAPbI_3_. CsFAPbIBr has a few of the same low-intensity peaks, but the pattern is dominated by the PbI_2_ (002) diffraction peak (2Θ ∼ 26°).^54^ By comparing the higher angle diffraction peaks, we can attribute the peaks between 2Θ ≃ 11°–13° at 250 °C to 4H-FAPbI3 for CsFAPbI and PbI_2_ for CsFAPbIBr. Though the film is very textured, causing the XRD pattern to be dominated by diffraction from PbI_2_, the β-perovskite is still present in both films (Figure S3). From the analysis of the XRD patterns, it can be seen that adding 17% bromine seems to reduce the range of temperatures where the β-perovskite phase is stable and switches the primary products of the phase transformation from δ-CsPbI_3_ and 4H-FAPbI_3_ to PbI_2_.

**Figure 2:**
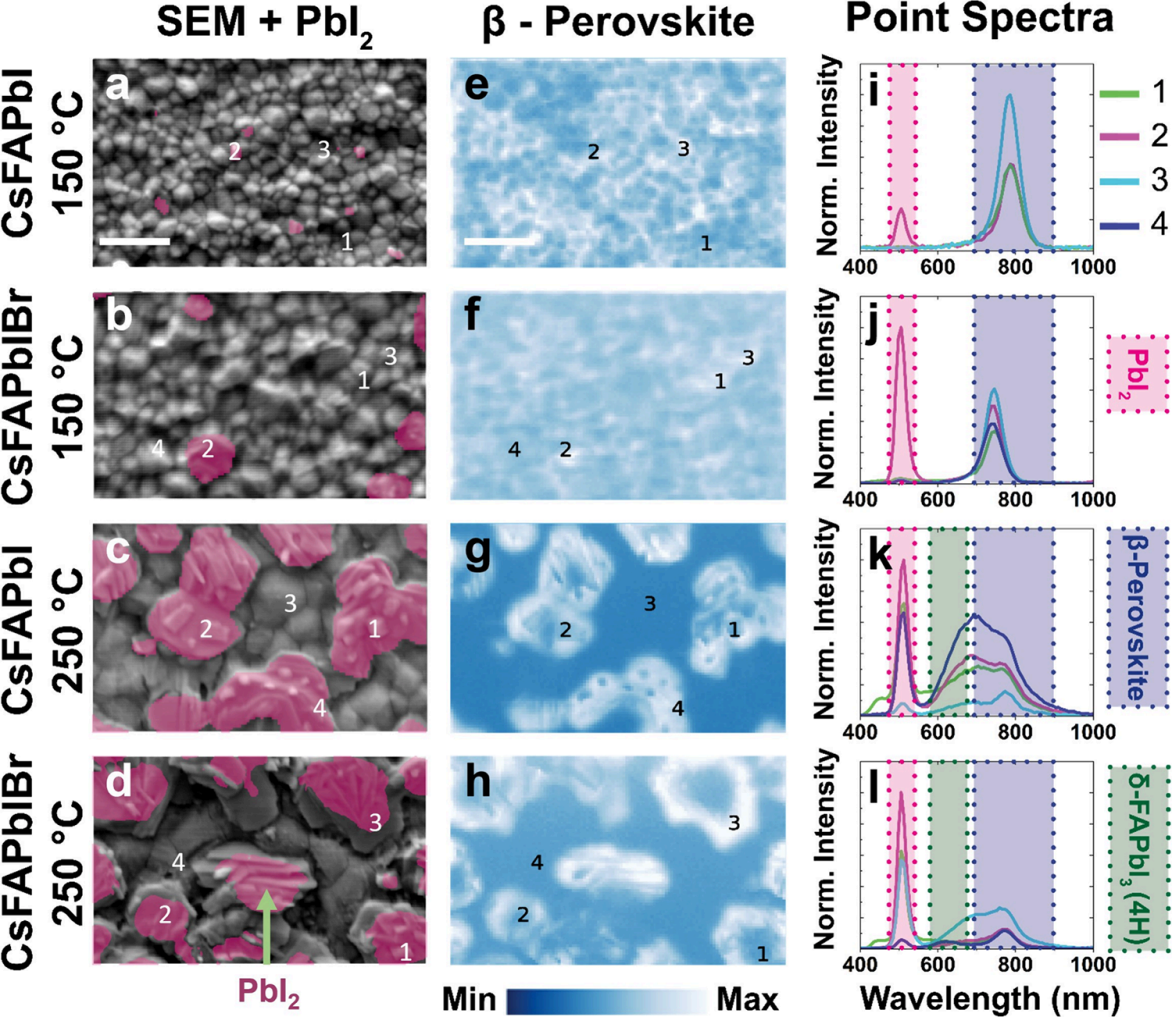
Cathodoluminescence SEM correlates morphology and optical properties. (a–d) SEM images with pink overlays indicating areas of PbI_2_ (Note S1)_._ Scale bar = 1 μm. (e–h) Intensity maps of perovskite emission (786 nm for CsFAPbI and 745 nm for CsFAPbIBr). Scale = 1 μm. (i–l) Point CL spectra from labeled spots 1–4 on corresponding SEM and perovskite intensity maps. Shaded areas correspond to characteristic PbI_2_, β-perovskite, and δ-FAPbI_3_ emission.

To correlate temperature-induced phase transformations and segregation with changes in morphology, we used CL-SEM mapping (Figure 2) on thin films fabricated using two different annealing temperatures: the standard used for solar cell fabrication (150 °C) and a temperature known to produce significant amounts of secondary phases (250 °C). CL-SEM connects morphological and optical properties by using a parabolic mirror to collect electron beam induced light emission from the thin film while simultaneously taking the SEM image.^56−58^ The emission spectra are collected at each pixel and can be plotted as a function of wavelength (Figure 2i–l) or can be used to create intensity maps of the emission at a specific wavelength (Figure 2e–h). PbI_2_ emission is centered at 505 nm,^47^ while emission from the perovskite ranges from 740 to 800 nm depending on local composition.^47,59^ Emission from δ-CsPbI_3_ is seen as broadband emission centered around 443 nm.^47,60,61^ The hexagonal-FAPbI_3_ phases (2H, 4H, and 6H) exhibit broadband emission in the range of ∼600–700 nm.^47,62^ The distance between the emission peaks of these key phases makes it possible to differentiate them and correlate structural, morphological, and optical changes. As a general note, the CL-SEM images show slight differences in secondary phase (δ-CsPbI_3_, 4H-FAPbI_3_, PbI_2_) composition and quantity compared to the XRD data in Figure 1. This is because CL-SEM is a surface technique that can spatially resolve CL depending on local phase differences that are exacerbated at the surface as the film is exposed to different environments. XRD is a bulk technique where preferential orientation or poor crystallinity could cause diffraction from the secondary phases to be below the detection limit. Full intensity maps for δ-CsPbI_3_, PbI_2_, and β-perovskite are shown in Figures S4 and S5.

To consider changes in morphology and correlate them with structural changes in XRD, Figure 2a–d shows regular SEM images with pink overlays to indicate areas that have been identified as PbI_2_ from their emission (Note S1). The amount of PbI_2_ present increases upon adding bromine and at a higher annealing temperature. At 250 °C, more PbI_2_ emission also correlates with larger grain sizes (Figure 2c,d). It is challenging to quantify the amount of PbI_2_ in both CsFAPbIBr and CsFAPbI by only examining the maps. However, the point CL spectra (Figure 2i–l) are normalized to the maximum emission from each sample to provide an idea of which species contribute most to the emission. For CsFAPbI at 250 °C, all selected points show emission from PbI_2_, β-perovskite, and 4H-FAPbI_3_ (Figure 2k), as would be expected from XRD (Figure 1a). Though the PbI_2_ emission is the highest, the emission from the β-perovskite and 4H-FAPbI_3_ phases are still quite significant. In contrast, after adding bromine, the emission from CsFAPbIBr at 250 °C (Figure 2l) is dominated by PbI_2_. This conclusion from CL-SEM further supports our hypothesis from the structural analysis that adding bromine shifts the degradation products from 4H-FAPbI_3_ to PbI_2_. In both CsFAPbI and CsFAPbIBr at 250 °C, there is also emission from the δ-CsPbI_3_ phase as is evidenced by the shoulder below 500 nm at point 1 in Figure 2k,l. The formation of the δ-CsPbI_3_ phase in both compositions at high temperatures is further supported by X-ray fluorescence (XRF) (Figures S6 and S7,Tables S1 and S2) and X-ray beam induced current (XBIC) mapping (Figure S8). High Cs areas in XRF have been shown to be the result of phase segregation of the Cs/FA cations that produce the low-current δ-CsPbI_3_ phase.^63,64^

To further examine the optical properties of the perovskite, filtered intensity maps of the β-perovskite emission are shown in Figure 2e–h and are supplemented by point spectra taken at the numbered points in Figure 2i–l. The selected filtered wavelength changes with the bandgap of the perovskite (786 nm for CsFAPbI and 745 nm for CsFAPbIBr). Both CsFAPbI and CsFAPbIBr have relatively homogeneous intensities across the β-perovskite emission maps at 150 °C, but by 250 °C they develop significant spatial heterogeneities in emission intensity. Larger grains on the surface have higher emission intensities but the same local composition as points deeper on the surface, as can be seen by comparing the point spectra at points 3 and 4 for CsFAPbI (Figure 2g,k) and CsFAPbIBr (Figure 2h,l). These point spectra have emission peaks at the same wavelengths but overall lower intensity due to variations in height of the film caused by secondary phases growing on the surface.^65^

As the annealing temperature increases from 150 to 250 °C, CsFAPbI (Figure 2k) loses the sharp characteristic perovskite peak around 780 nm^47,59^ and instead broadens into a wide peak spanning 600–800 nm. This broad peak is indicative of the formation of hexagonal-FAPbI_3_ phases (also known as 2H, 4H, and 6H).^66,67^ However, we can conclude that this is likely the 4H-FAPbI_3_ phase in accordance with our XRD analysis (Figure 1). The center of the remaining perovskite emission undergoes an overall blue shift (787 to 772 nm) that is likely due to the loss of organic as FA^+^ and I^–^, creating Cs-rich local areas that have a bandgap that causes emission at lower wavelengths.^13^ For CsFAPbIBr at 250 °C (Figure 2h), there is some peak broadening that can be attributed to 4H-FAPbI_3,_ but the emission is dominated by PbI_2_. The β-perovskite emission is red shifted (745 to 772 nm) due to the loss of FA^+^ and Br^–^ with increasing temperature. The loss of FA^+^ and Br^–^ creates I^–^ rich areas that have a bandgap that causes emission at higher wavelengths.^13^ Optical and morphological mapping using CL-SEM confirms the changes in phase transformation pathway with the addition of bromine suggested by XRD. CsFAPbI forms δ-CsPbI_3_ and 4H-FAPbI_3_, but the addition of bromine in CsFAPbIBr shifts secondary phases toward PbI_2_. Shifts in the wavelength of the β-perovskite emission peak suggest the loss of halides and/or the organic could be associated with these transformations.

To dive into the thermodynamic mechanisms behind phase transformations influenced by mixing halides, we used density functional theory (DFT). Initially, we examined pristine bulk perovskites focusing on three distinct model systems: CsFA_3_Pb_4_I_12_ (eq 1a), CsFA_3_Pb_4_I_11_Br (eq S1), and CsFA_3_Pb_4_I_10_Br_2_ (eq 2). These model systems are designed to demonstrate the effect of a progressive increase in bromine on the thermodynamics of phase transformations of mixed cation perovskites. Theoretical lattice parameters are found in Table S3. Guided by the experimental XRD measurements, the products were discerned to be distributed stoichiometrically among δ-CsPbI_3_, PbI_2_, FABr, and FAI (eq 1a) with subsequent thermodynamically favored recombination of FAI and PbI_2_ into 4H-FAPbI_3_ (eq 1b). Notably, all reactions exhibited endothermic characteristics, but a subtle uptick in exothermicity (and thus thermodynamic favorability) was seen with increasing bromine (eqs 1a and 2). However, we expected a more prominent influence from the increased bromine content on the phase transformations based on the experimental results.
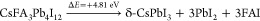
1a
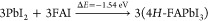
1b
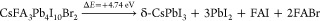
2Consequently, we shifted our focus to vacancy formation (V_I_ and V_Br_) as a possible mechanism for facilitating phase transformations across the models. Specifically, phase transformations in the presence of V_I_ were assessed for CsFA_3_Pb_4_I_12_ (eq 3) while both V_I_ and V_Br_ were scrutinized for the bromine-inclusive perovskites (eq 4 and eq S2).
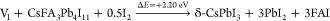
3
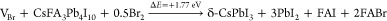
4

Relative to the pristine systems, the presence of a halide vacancy showed a marked increase in favorability of phase transformation from the β-perovskite to δ-CsPbI_3_, PbI_2_, and FAI/FABr, with a reduction of energy of formation greater than ∼2 eV. Lower formation energy indicates a greater likelihood of degradation. In systems with halide vacancies, the formation energy of these secondary phases in the presence of bromine decreased by 0.43 eV compared to CsFA_3_Pb_4_I_12_ (eqs 3 and 4). This is in contrast to the pristine perovskites, where the decrease was only 0.07 eV with and without bromine (eqs 1a and 2). This data emphasizes the increased susceptibility of bromine-containing perovskites to phase transformations, particularly when vacancies are present and under elevated temperatures, aligning with our experimental findings.

With the knowledge that degradation often initiates from the surface of a material, we also investigated the perovskite surface using DFT. The perovskite surface shows a slight destabilization of the surface energy with the inclusion of bromine, indicating an increased potential for the formation of secondary phases. This destabilization becomes more pronounced when a vacancy is introduced (Figure S9). This underscores the pivotal role of vacancy formation—especially prominent in bromine-rich systems due to the loss of FABr—in driving the degradation of mixed halide perovskites.

**Figure 3:**
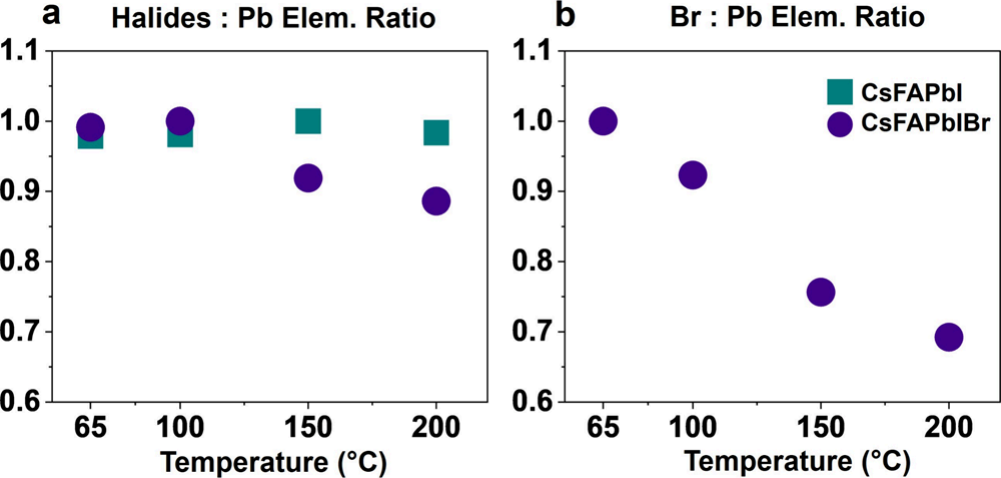
Normalized XPS elemental ratios show halide loss with increasing temperature. (a) Combined ratio of halides (I, Br) to Pb and (b) Br to Pb from integrated area under the curve from XPS to show loss of halides from surface of thin film as annealing temperature increases. The maximum value for each composition is normalized to 1.

Based on the predictions by DFT, we investigated changes in the surface composition of CsFAPbI and CsFAPbIBr films via XPS to understand if halides are lost with annealing. Loss of halides during annealing would lead to the formation of vacancies and destabilize the β-perovskite phase. By fitting the characteristic peaks for each element and integrating the area under the curve, we quantified changes in the elemental composition. Full fits are found in Figures S10 and S11. To compare production of halide vacancies between compositions and annealing temperatures, we calculated the ratio of the areas under the curve for halides (I, Br) to lead for both compositions (Figure 3a) and bromine to lead for CsFAPbIBr (Figure 3b). The halide to lead elemental ratio is relatively constant for CsFAPbI but decreases above 100 °C for CsFAPbIBr. This loss of halides relative to lead in CsFAPbIBr supports the conclusions from DFT that adding bromine facilitates phase transformations in the presence of halide vacancies. Looking specifically at the bromine to lead ratio (Figure 3b), the amount of bromine relative to the lead in the film decreases by more than 30% as the annealing temperature increases from 65 to 200 °C. This loss of bromine relative to lead aligns with past works and the conclusions from DFT that suggest CsFAPbIBr loses material in the form of FABr at as low as 100 °C.^18,35^ However, the exact chemical state (i.e., FABr vs other forms of Br) of material lost is difficult to determine and quantify via nitrogen XPS (Figure S12), and more research will be required to understand it. However, the XPS shows the conclusive loss of halides relative to lead at lower temperatures in CsFAPbIBr when compared to CsFAPbI as suggested by DFT. Additional X-ray fluorescence mapping (XRF) elemental maps, X-ray beam induced current (XBIC) maps, and calculated molar ratios showing the same trends in halide loss are found in the Supporting Information (Figures S6–S8, Tables S1 and S2). Adding bromine causes production of halide vacancies at lower temperatures, via the likely loss of FABr, which reduces thermodynamically favorable recombination into 4H-FAPbI_3_, shifting degradation toward PbI_2_ in CsFAPbIBr.

**Figure 4:**
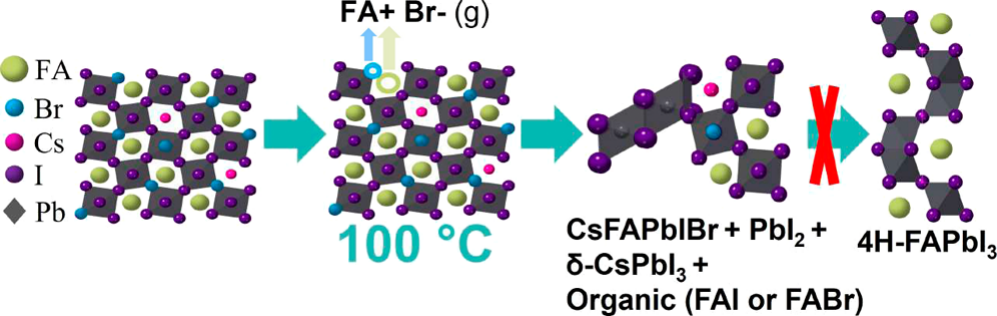
Schematic of shift in products in the presence of bromine with DFT calculations. Upon the production of halides via hypothesized volatilization of FABr beginning at 100 °C, the thermodynamically favorable recombination of PbI_2_ and FAI to 4H-FAPbI_3_ is limited based purely on stoichiometry. Thus, upon adding bromine, degradation products shift toward PbI_2_.

The schematic in Figure 4 shows the changes caused by adding bromine both in the pristine perovskite and after the creation of V_Br_ all underpinned by our DFT calculations (shown with the equations in Figure S13). As shown in eqs 1a and 2, in CsFAPbI and CsFAPbIBr, phase transformations initiate with a breakdown into δ-CsPbI_3_, FAI, FABr, and PbI_2_. Subsequently, it is highly thermodynamically favorable for PbI_2_ and FAI to combine into the 4H-FAPbI_3_ phase (eq 1b). However, in the case of CsFAPbIBr, halide vacancies will form at a temperature as low as 100 °C, likely due to the volatilization of organic FABr (FA^+^, Br^–^). These halide vacancies significantly lower the energy of formation (eqs 3 and 4), thus reducing the thermal stability with bromine. After the likely volatilization of FA^+^ and Br^–^, less of the organic material is available to recombine into the 4H-FAPbI_3_ phase from a purely stoichiometric perspective. This shifts the degradation products toward more PbI_2_ in the presence of bromine. It is also worth noting that above 150 °C, even for CsFAPbI, we see a rise in PbI_2_ levels. This is due to the volatilization of the organic (FA^+^, I^–^), which begins around 160 °C,^35^ causing degradation into PbI_2_ via the same mechanism, but at higher temperatures than in the presence of bromine (Figure S12). This reinforces the detrimental impact of adding bromine on the thermal stability.

**Figure 5:**
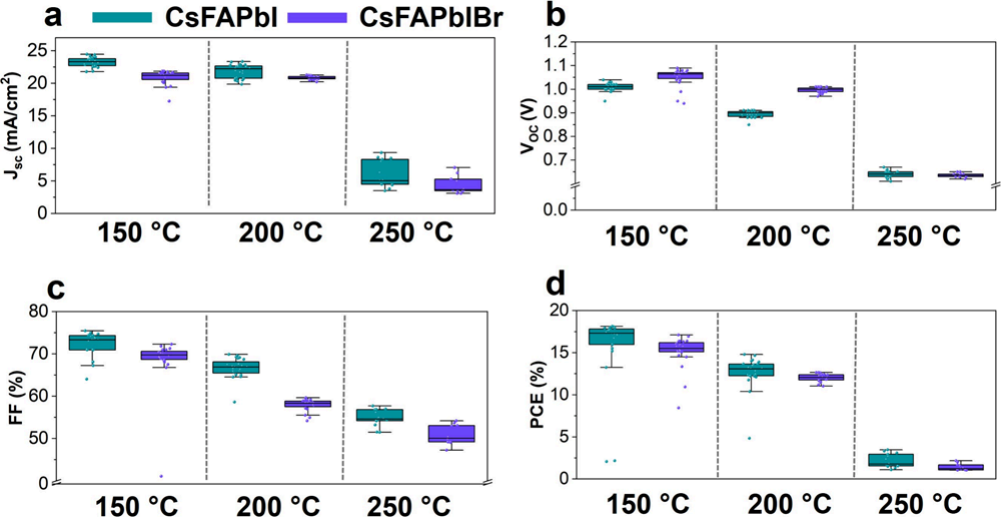
Device performance as a function of composition and temperature: (a) Short-circuit current density (*J*_SC_), (b) open-circuit voltage (*V*_OC_), (c) fill factor (FF), and (d) power conversion efficiency (PCE) from reverse scans of n–i–p devices.

To understand the effect that differences in phase transformation products with and without bromine have on photovoltaic performance, n–i–p solar cells were fabricated and tested (Figure 5) with the perovskite film annealed at different temperatures. As expected, due to the increasing presence of secondary phases, performance decreases with increasing annealing temperature and adding bromine. The higher *V*_OC_ in the presence of bromine can be attributed to the increased bandgap.^68^ Notably, both compositions show measurable performance after 250 °C annealing, which indicates the potential for photovoltaic performance, even with significant amounts of degradation and secondary phases present. Full device parameters from forward and reverse bias can be found in Figure S14. Even for highly degraded samples, CsFAPbI performs slightly better than does CsFAPbIBr. While the difference in performance is very small, one could speculate that the secondary phase 4H-FAPbI_3_ in CsFAPbI has higher mobilities than those in CsFAPbIBr (mainly PbI_2_) due to an increase in corner sharing for the 4H-FAPbI_3_ phase. This could in turn translate into an improved FF, which we see in Figure 5c. To understand the hysteresis dynamics in devices, we calculated the hysteresis index and conducted IMPS measurements (Figure S15). The hysteresis index increases with annealing temperature, as is expected with increased production of defects. However, hysteresis is a convolution of many phenomena, including morphology and band alignment, among others.^69^ IMPS shows a shift toward lower frequencies with increasing annealing temperatures. This indicates slower ionic transport, which enables more hysteresis in a current–voltage measurement when the scan rate is kept constant. Both techniques indicate changes in device performance after phase transformations, possibly due to changes in ion migration caused by vacancies, but the influence of other confounding factors cannot be ignored.

## Conclusions

Through a combination of structural characterization, morphological and optical mapping, XPS, and first-principles calculations, we have unraveled a mechanism that enables different types of phase transformations as a function of chemistry and annealing temperature. We show that replacing 17% of iodine with bromine in Cs_0.17_FA_0.83_PbI_3_ reduces thermal stability and shifts the products of phase transformation from δ-CsPbI_3_ and 4H-FAPbI_3_ toward PbI_2_. Previously established low-temperature production of halide vacancies in the presence of bromine are shown to accelerate new phase transformation mechanisms and prevents recombination of resultant secondary phases into 4H-FAPbI_3_, instead causing a shift toward PbI_2_. These differences, in which secondary phases are present, have been shown to affect solar cell performance and likely have much wider impacts on hysteresis, charge transport, and ion migration. This work has implications for the current research environment to emphasize the importance of the effect of annealing temperature on crystallographic properties and phase transformations in mixed-cation and mixed-halide systems and sets the stage for future studies on the effects of differing secondary phases on long-term solar cell performance and stability.

## Supporting Information Available

XRD patterns, CL-SEM maps, XRF/XBIC maps, XPS elemental ratios and fits, and device performance characterization; additional DFT calculations and schematics (PDF)
