# Supplementary material for: Bromine Incorporation Affects Phase Transformations and Thermal Stability of Lead Halide Perovskites
Source: J Am Chem Soc. 2024 Jun 27;146(27):18576–85. doi: 10.1021/jacs.4c04508 (PMC11240253; doi:10.1021/jacs.4c04508)
Supplement: Supplementary file 1 — ja4c04508_si_001.pdf [file ja4c04508_si_001.pdf]

Supporting Information

Bromine Incorporation Affects Phase Transformations and Thermal Stability of Lead Halide Perovskites

Diana K. LaFollette[1], Juanita Hidalgo[1], Omar Allam[2], Jonghee Yang[3,4], Austin Shoemaker[1], Ruipeng Li[5], Barry Lai[6], Benjamin Lawrie[7,8], Sergei Kalinin[9,10], Carlo A.R. Perini[1], Mahshid Ahmadi[4], Seung Soon Jang[1], Juan-Pablo Correa-Baena[1,11*]

[1]School of Materials Science and Engineering, Georgia Institute of Technology, North Ave NW, Atlanta, GA 30332, USA
[2]Woodruff School of Mechanical Engineering, Georgia Institute of Technology, North Ave NW, Atlanta, GA 30332, USA
[3]Department of Chemistry, Yonsei University, Seoul 03722, Republic of Korea
[4]Institute for Advanced Materials and Manufacturing Department of Materials Science and Engineering, University of Tennessee Knoxville, Knoxville, TN 37996, USA
[5]National Synchrotron Light Source II, Brookhaven National Lab, Upton, NY, 11973, USA
[6]Advanced Photon Source, Argonne National Laboratory, Lemont, IL, 60439, USA
[7]Center for Nanophase Materials Sciences, Oak Ridge National Laboratory, Oak Ridge, TN 37831, USA
[8]Materials Science and Technology Division, Oak Ridge National Laboratory, Oak Ridge, TN, 37831, USA
[9]Department of Materials Science and Engineering, University of Tennessee, Knoxville, TN, 37996 USA
[10]Physical Sciences Division, Pacific Northwest National Laboratory, Richland, WA 99352 USA
[11]School of Chemistry and Biochemistry, Georgia Institute of Technology, North Ave NW, Atlanta, GA 30332, USA

*Corresponding author: JPCB jpcorrea@gatech.edu

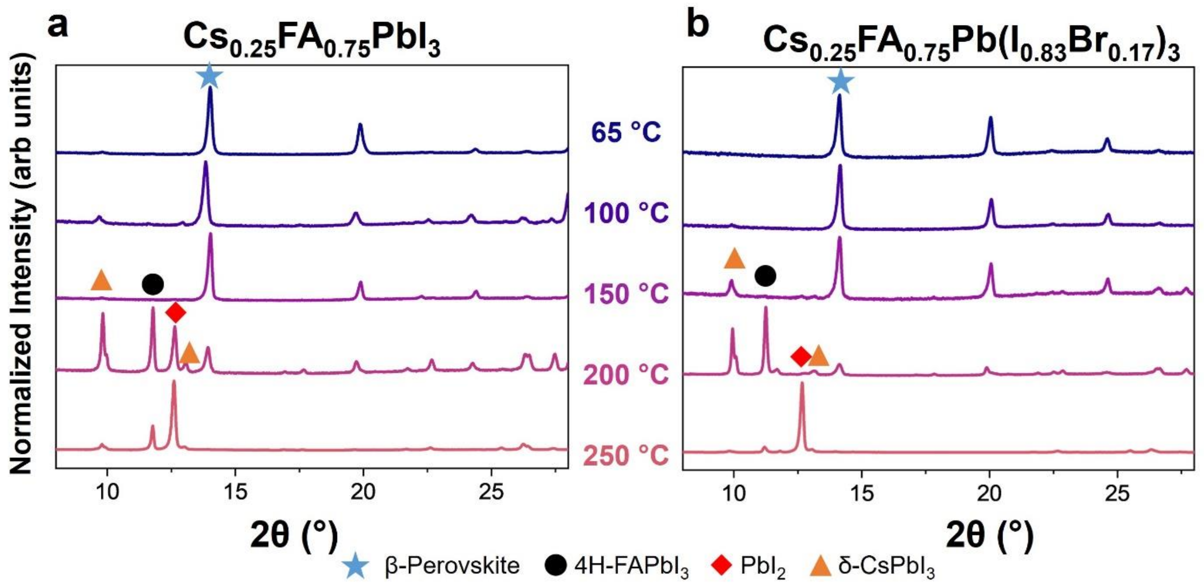

Figure S1. Normalized XRD Patterns of Exact Compositions used for DFT Calculations. a) $Cs_{0.25}FA_{0.75}PbI_3$ b) $Cs_{0.25}FA_{0.75}Pb(I_{0.83}Br_{0.17})_3$. XRD patterns of 25%-Cs compositions used in DFT show that the same trends apply for $Cs_{0.25}FA_{0.75}PbI_3$ and $Cs_{0.25}FA_{0.75}Pb(I_{0.83}Br_{0.17})_3$ as $Cs_{0.17}FA_{0.83}PbI_3$ and $Cs_{0.17}FA_{0.83}Pb(I_{0.83}Br_{0.17})_3$. $Cs_{0.25}FA_{0.75}Pb(I_{0.83}Br_{0.17})_3$ has reduced thermal stability compared to $Cs_{0.25}FA_{0.75}PbI_3$ as evidenced by the appearance of the δ-$CsPbI_3$ and 4H-$FAPbI_3$ peaks appearing as low as 150 °C. The final phase transformation products of $Cs_{0.25}FA_{0.75}Pb(I_{0.83}Br_{0.17})_3$ are dominated by PbI_2, while without bromine, there is still substantial influence from the δ-$CsPbI_3$ and 4H-$FAPbI_3$ peaks.

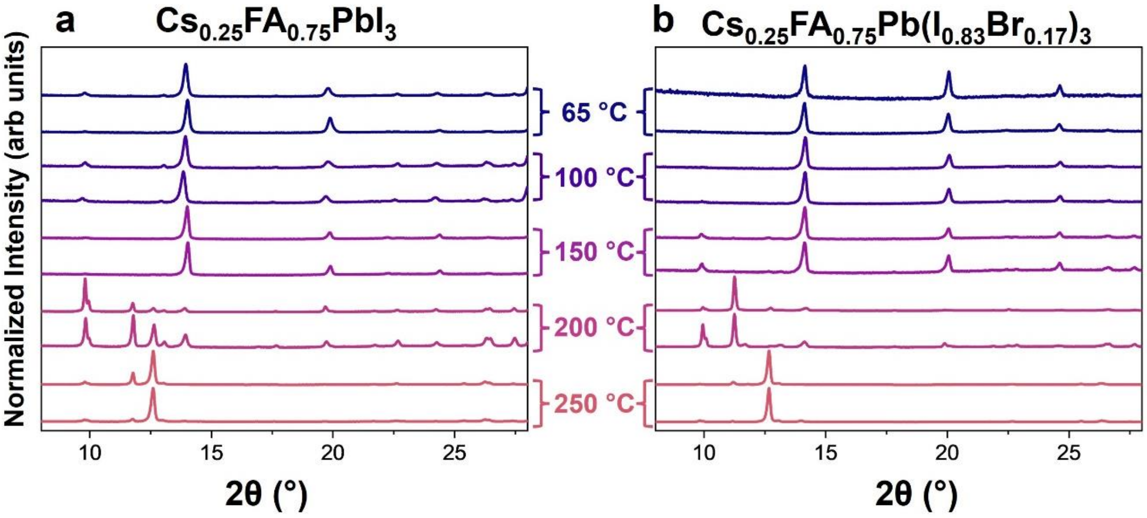

Figure S2. Normalized XRD Patterns Showing Reproducibility Across Duplicates. a) $Cs_{0.25}FA_{0.75}PbI_3$ b) $Cs_{0.25}FA_{0.75}Pb(I_{0.83}Br_{0.17})_3$. Duplicate samples show that phase transformations are consistent in phases present across compositions, even if there is slight variation in quantity.

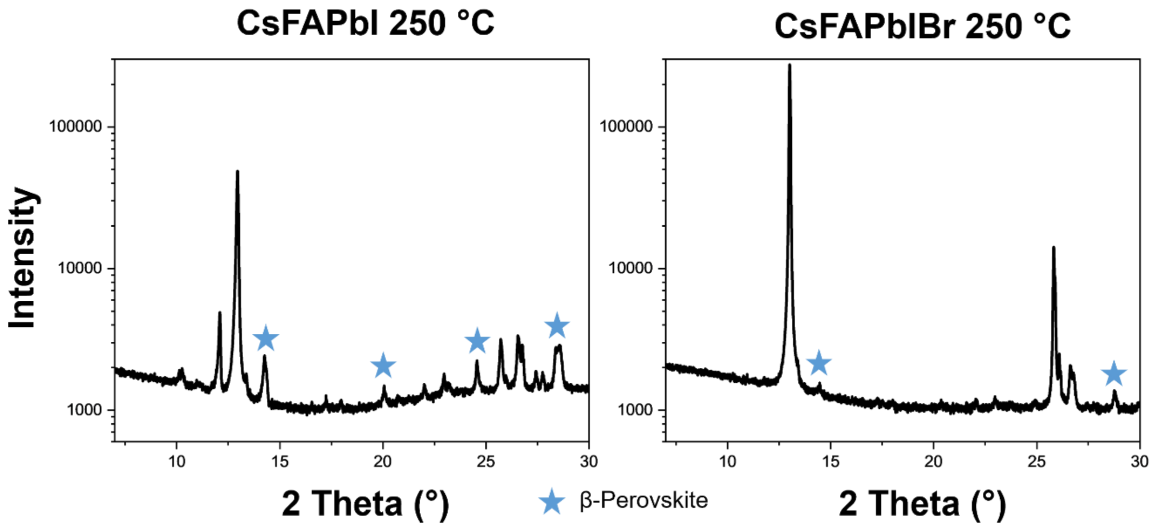

Figure S3. Log scale XRD patterns of CsFAPbI and CsFAPbIBr annealed at 250 °C to show the presence of the β-perovskite peak even at these high temperatures, though the diffraction patterns are dominated by textured secondary phases.

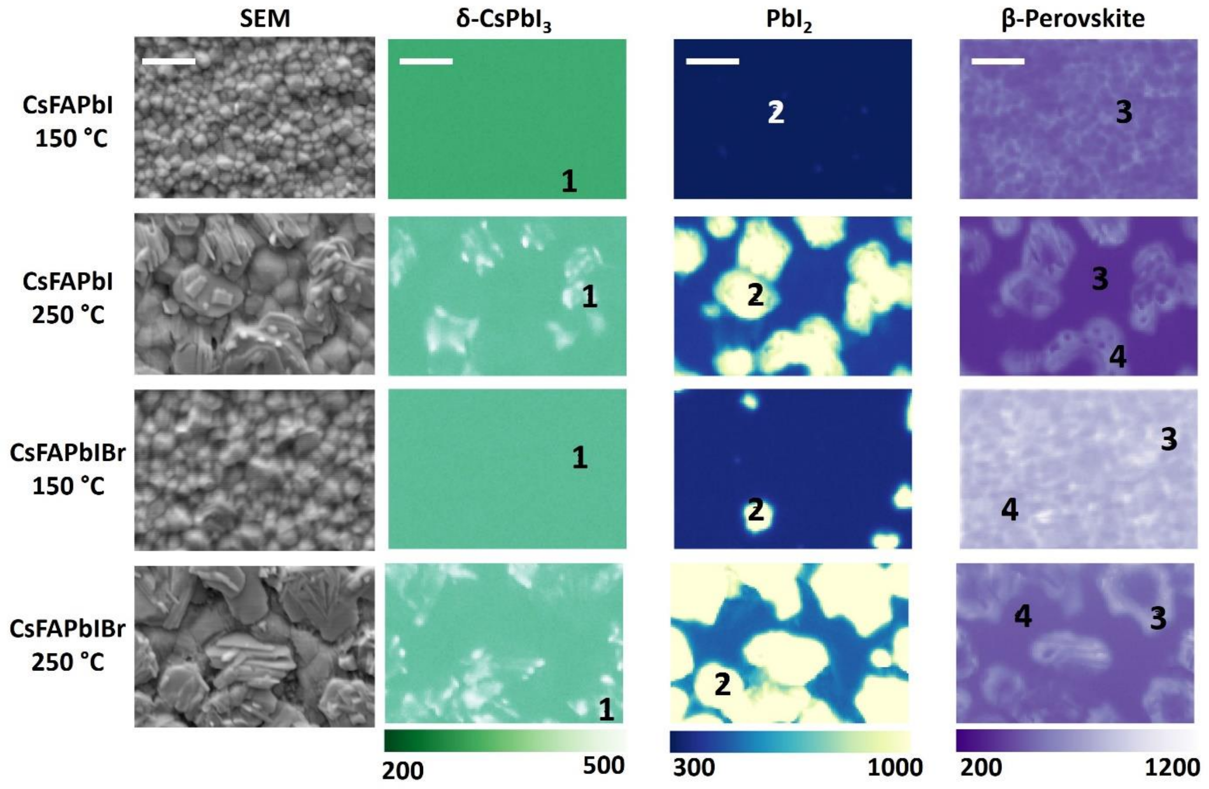

Figure S4. Complete Intensity Maps of CL-SEM. Column 1) SEM images **Column 2)** Intensity maps of characteristic δ-$CsPbI_3$ emission (443 nm). **Column 3)** Intensity maps of characteristic PbI_2 emission (505 nm). **Column 4)** Intensity maps of characteristic perovskite emission (780 nm for CsFAPbI, 745 nm for CsFAPbIBr). All scale bars equal 1 µm.

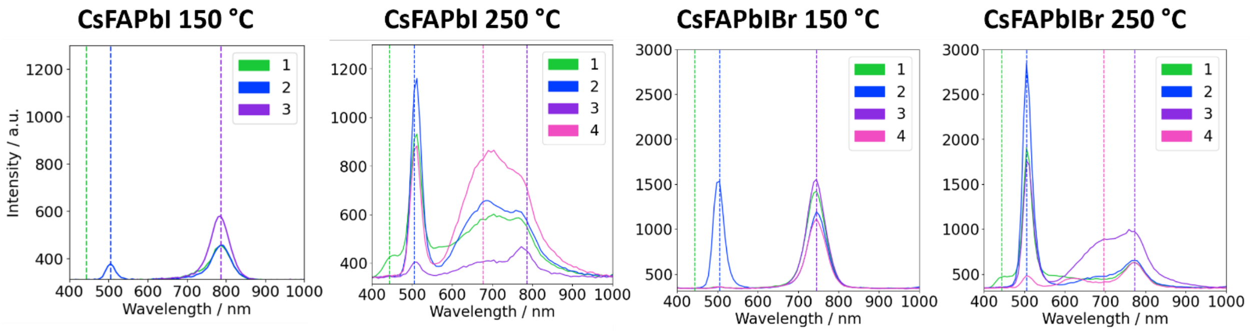

Figure S5. Point Spectra Corresponding to Spots Indicated on Figure S4.

Spots 3 and 4 on CsFAPbI and CsFAPbIBr 250 °C are chosen to show that even though there are differences in intensity due to surface roughness, the relative composition of the emission is the same. A spot on the grain is compared to a spot on the deeper surface, and though the deeper surface is lower emission, the location of peaks and their intensities relative to one another are the same. I.e. comparing CsFAPbI 250 °C spots 3 and 4, 3 is lower intensity than 4 but both have a broad perovskite emission from β-perovskite and 4H-$FAPbI_3$ with also some emission from PbI_2.

Note S1: Threshold Determination for Pink PbI_2 Overlay in Figure 2.

Intensity maps shown in Figure 2 were converted to black (no emission) and white (emission) using Adobe Illustrator. White areas were then extracted, and the black background removed before turning to pink and adding layer in overlay on top of SEM image.

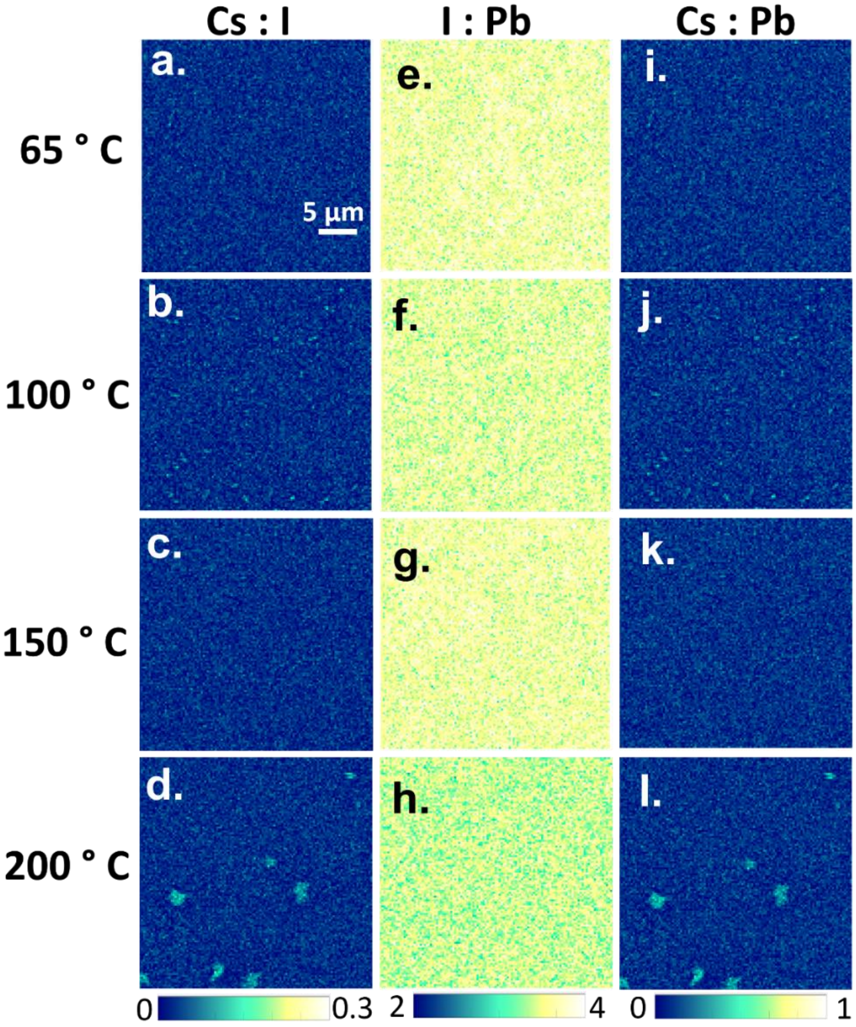

Figure S6. XRF Maps of Elemental Ratios for Varying Annealing Temperatures (CsFAPbI).

Table S1. Average elemental ratios for CsFAPbI XRF Maps shown in Figure S9.

Mean	I : Pb	Cs : I	Cs : Pb
**65 °C**	3.56	0.028	0.100
**100 °C**	3.49	0.028	0.097
**150 °C**	3.54	0.028	0.100
**200 °C**	3.38	0.029	0.099

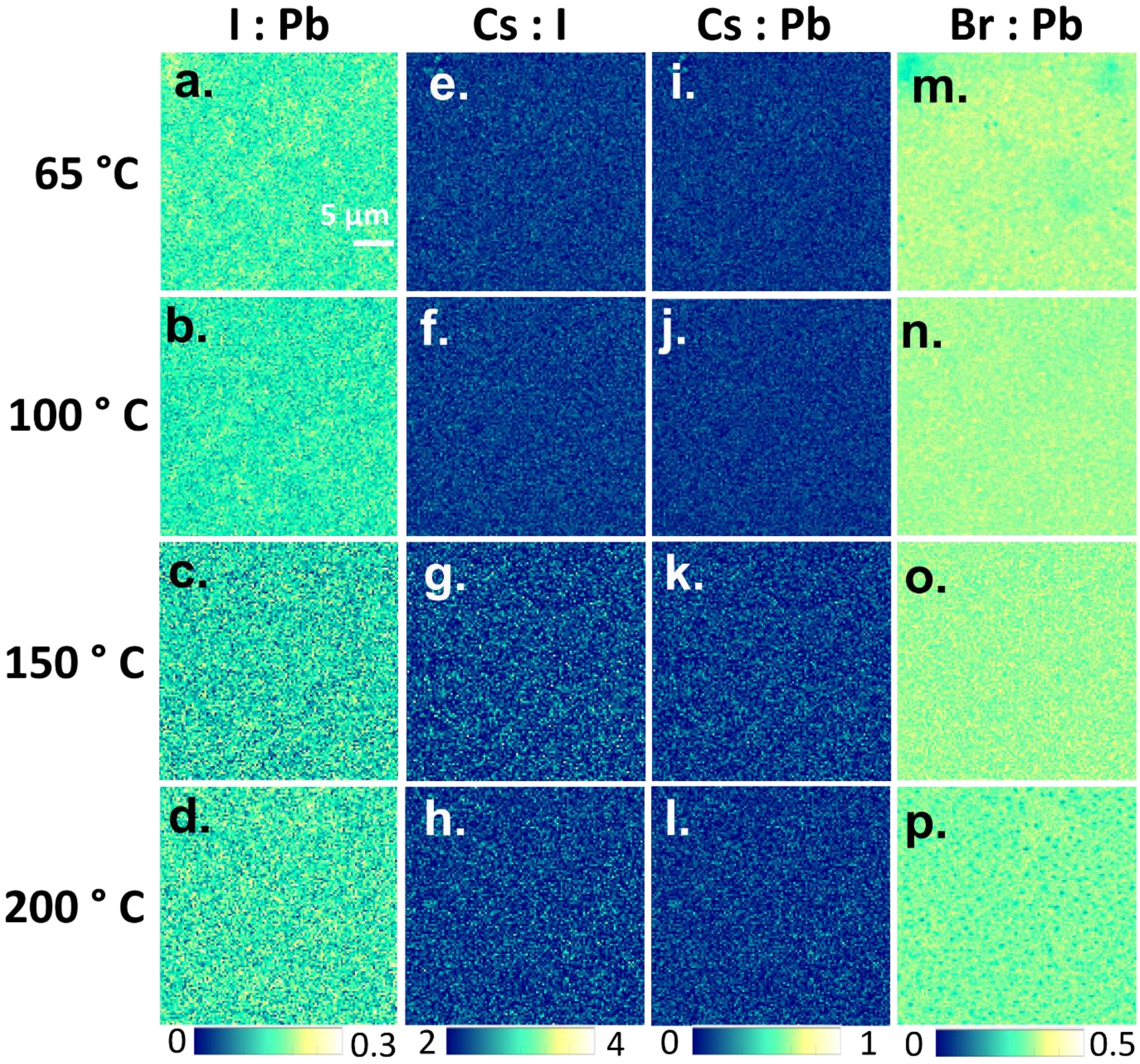

Figure S7. XRF Maps of Elemental Ratios for Varying Annealing Temperatures (CsFAPbIBr)

Table S2. Average elemental ratios for CsFAPbIBr XRF Maps (Figure S9)

Mean	I : Pb	Cs : I	Cs : Pb	Br : Pb	Br : I
**65 °C**	3.05	0.035	0.106	0.328	0.106
**100 °C**	2.97	0.035	0.105	0.326	0.108
**150 °C**	2.86	0.043	0.120	0.322	0.192
**200 °C**	2.98	0.038	0.111	0.303	0.161

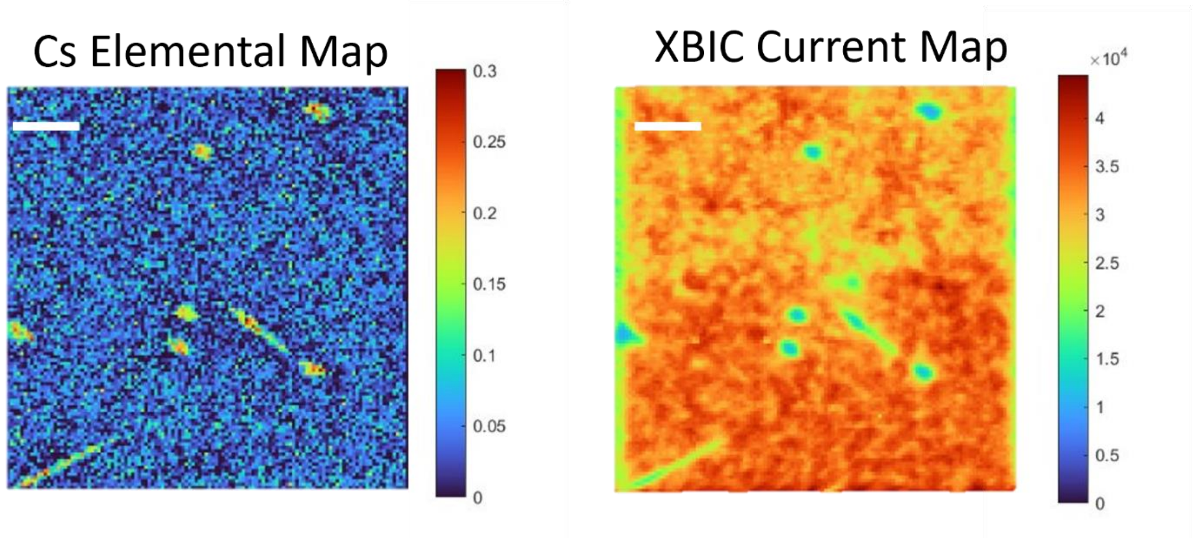

Figure S8. Sample X-ray beam induced current mapping showing that areas of high Cs content correspond to low current. Areas with low Cs and high current indicate the presence of δ-$CsPbI_3$. Scale bar is 5 μm.

Equations S1-S2) Additional thermodynamics of degradation reactions.

$$(S1)\ CsFA_3Pb_4I_{11}Br \xrightarrow{\Delta E=\ +4.76\ eV} \delta - CsPbI_3 + 3PbI_2 + 2FAI + FABr$$

$$(S2)\ V_I +\ CsFA_3Pb_4I_9Br_2 + 0.5I_2 \xrightarrow{\Delta E=\ +1.95\ eV} \delta - CsPbI_3 + 3PbI_2 + FAI + 2FABr$$

Table S3. DFT calculated lattice parameters and angles with changing composition.

Perovskite	a (Å)	b (Å)	c (Å)	α °	β °	γ °
$CsPbI_3$	8.904	8.904	12.597	90.000	90.000	90.000
$Cs_{0.25}FA_{0.75}PbI_3$	8.999	8.790	12.499	88.137	88.508	91.201
$Cs_{0.25}FA_{0.75}PbI_{2.75}Br_{0.25}$	9.045	8.741	12.339	88.060	88.288	90.946
$Cs_{0.25}FA_{0.75}PbI_{2.5}Br_{0.5}$	9.007	8.789	12.161	88.124	88.271	90.798

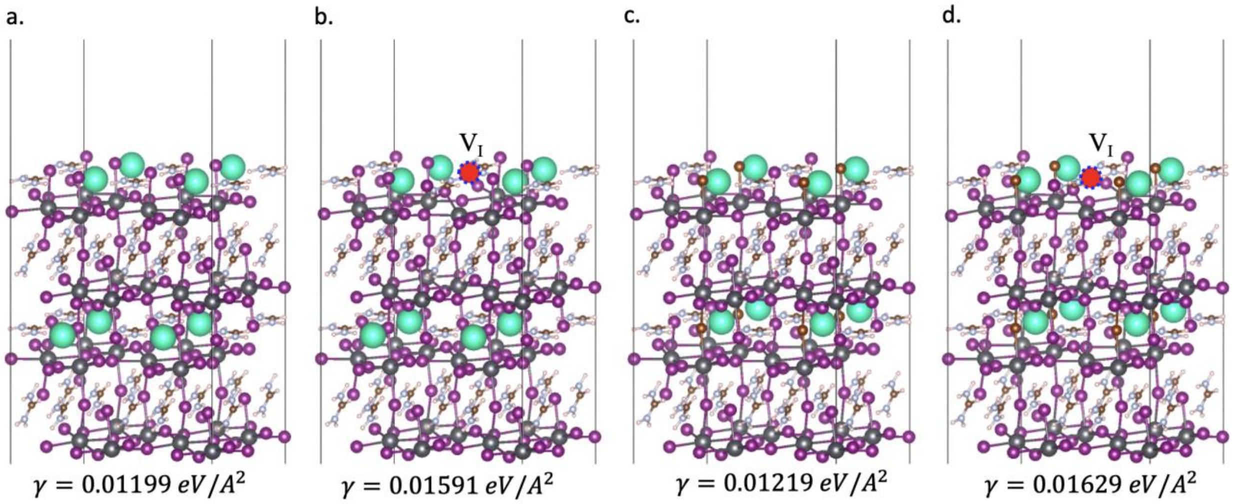

Figure S9. Evolution of perovskite surfaces. (a) I-only pristine surface. (b) I-only surface with V_I (iodine vacancy). (c) Mixed I-Br pristine surface. (d) Mixed I-Br with V_I. The formation of the vacancy induces a more significant increase in surface energy than the presence of Br alone, underscoring the substantial destabilizing effect of the vacancy on the perovskite surface.

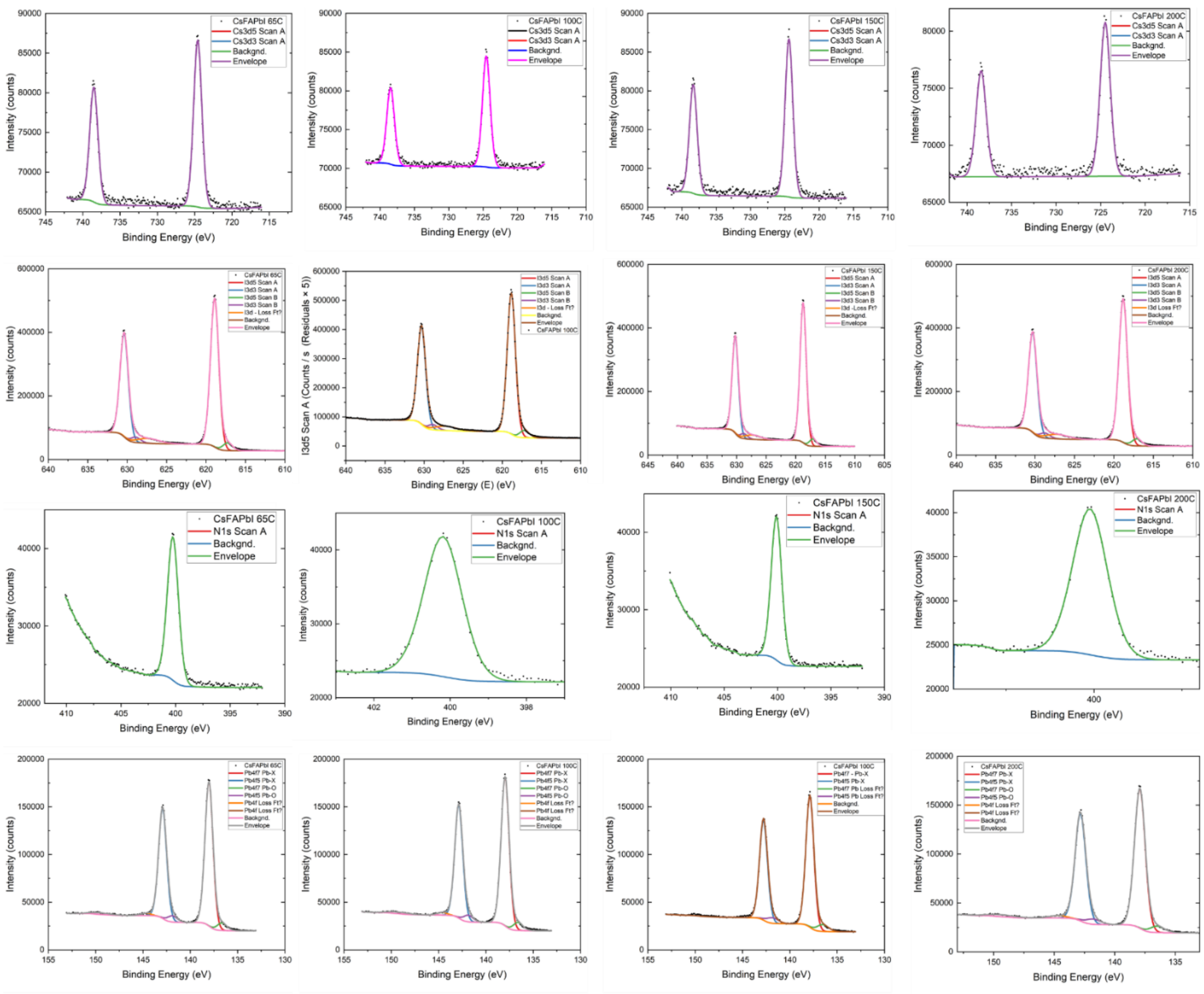

Figure S10. XPS Fits for CsFAPbI Used to Calculate Ratios

Loss features were used to correct background but are not included in ratios.

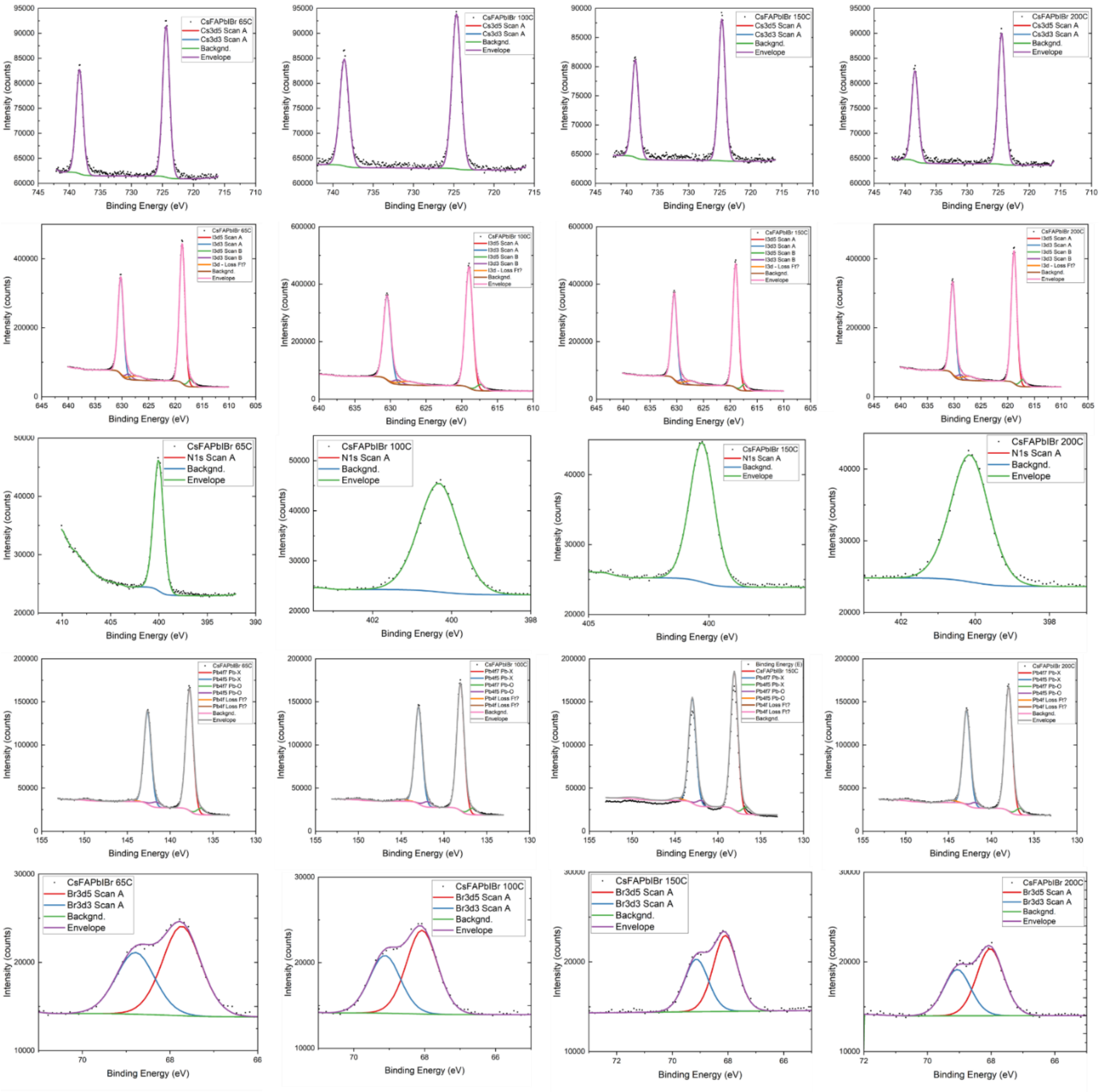

Figure S11. XPS Fits for CsFAPbIBr Used to Calculate Ratios

Loss features were used to correct background but are not included in ratios.

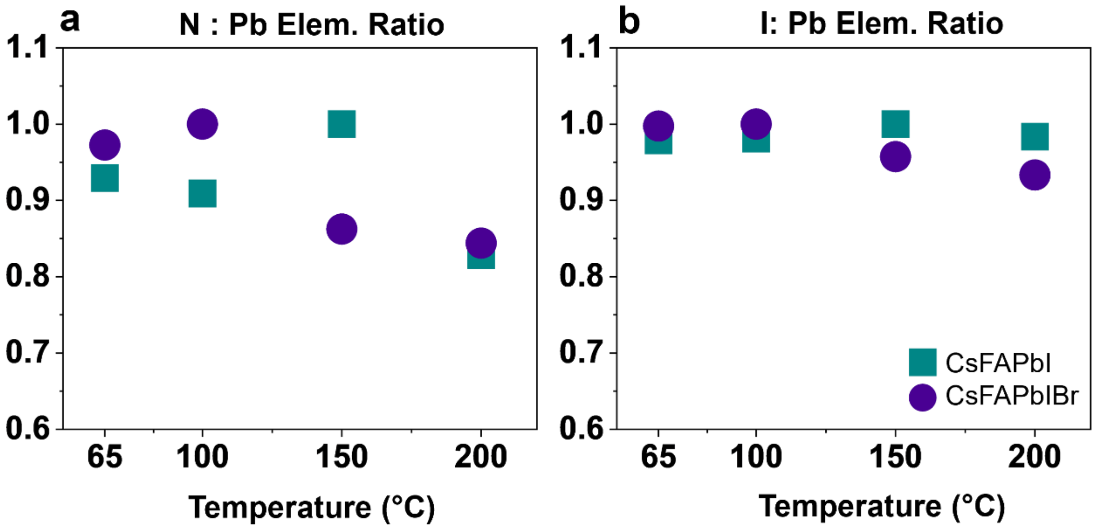

Figure S12. XPS Elemental Ratios with Varying Annealing Temperatures. a) N : Pb (b) I : Pb

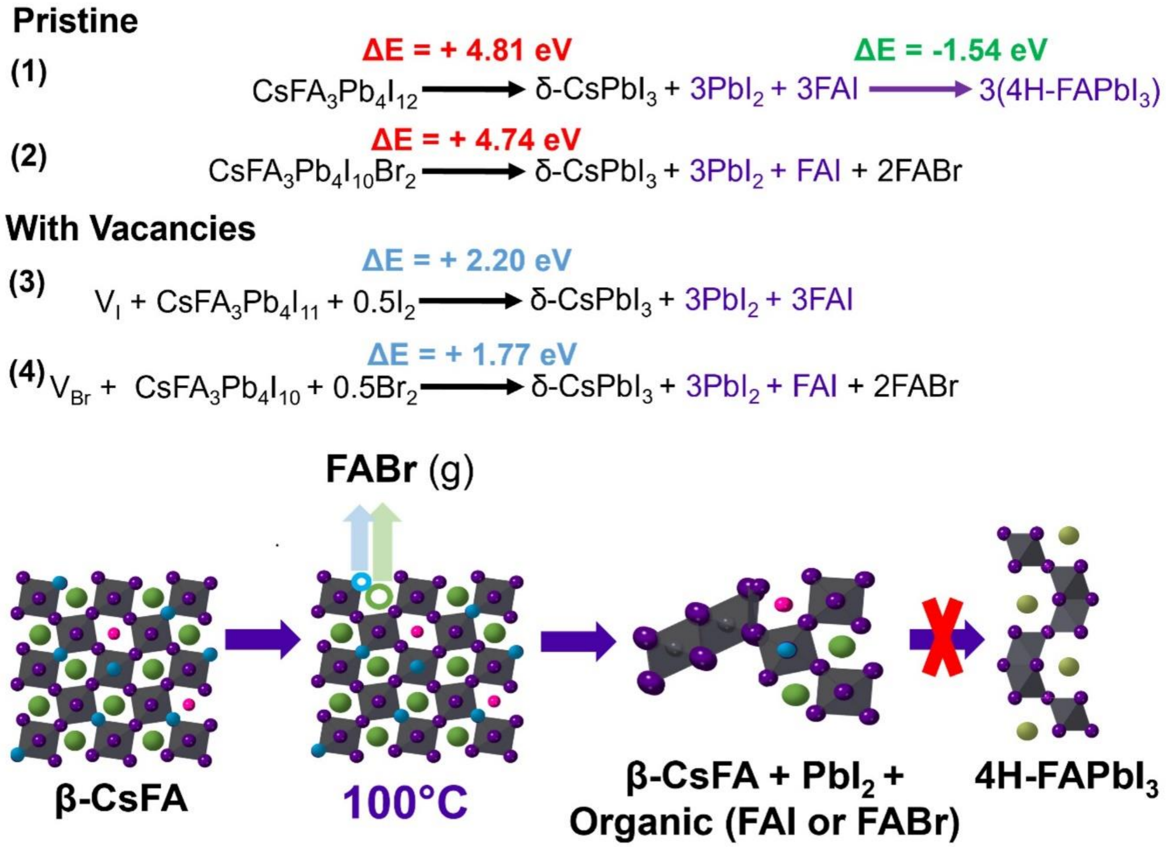

Figure S13. Schematic of Degradation Shift in Presence of Bromine with DFT Calculations. **(1)** Energy of degradation for pristine CsFAPbI illustrating the thermodynamic favorability of FAI and PbI_2 to recombine into 4H-$FAPbI_3$. **(2)** Energy of degradation of pristine CsFAPbIBr. **(3)** Degradation energy of CsFAPbI with an iodine vacancy. **(4)** Degradation energy of CsFAPbIBr with a bromine vacancy.

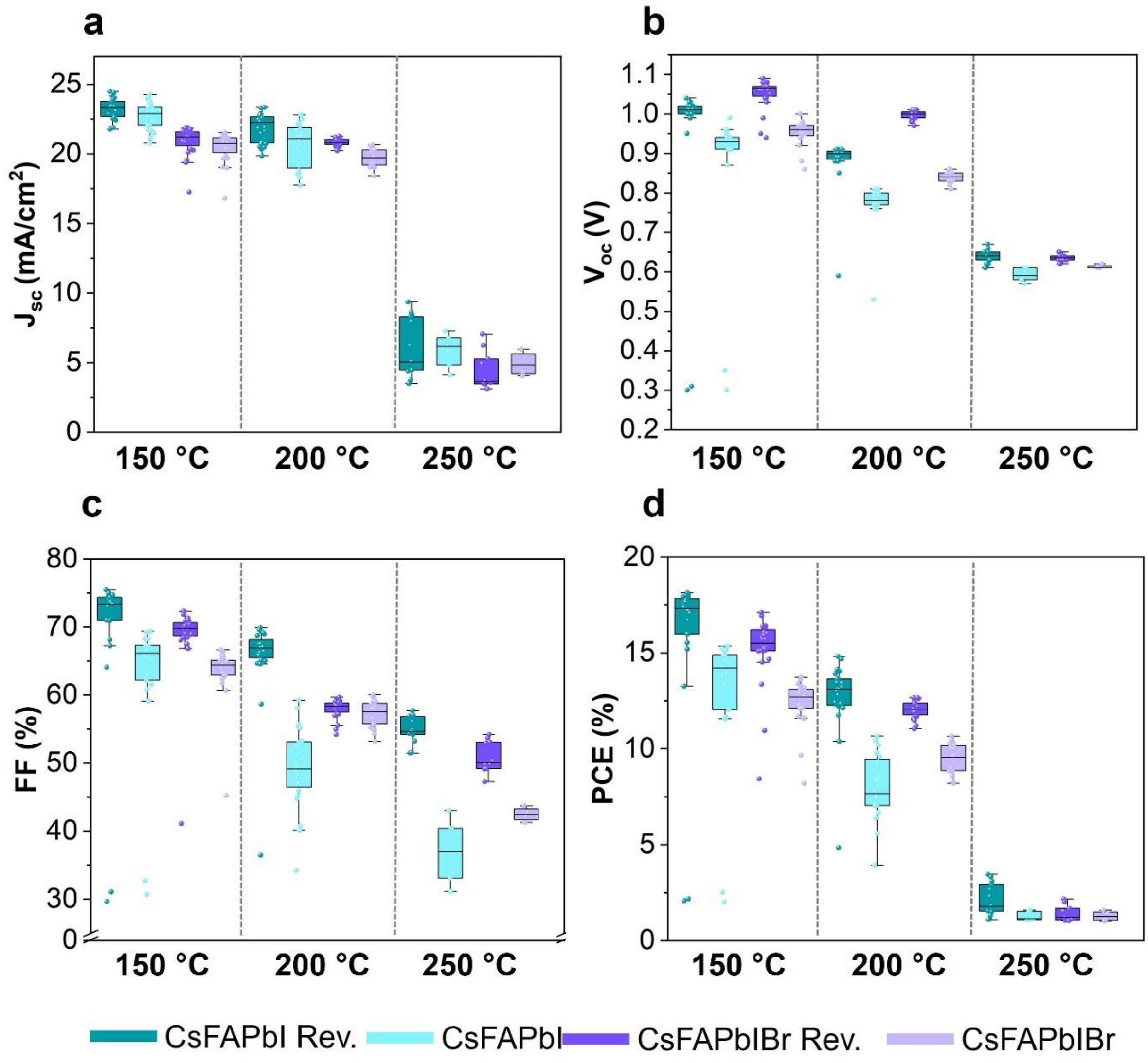

Figure S14. Forward and Reverse Device Data for Both Compositions and Annealing Temperatures

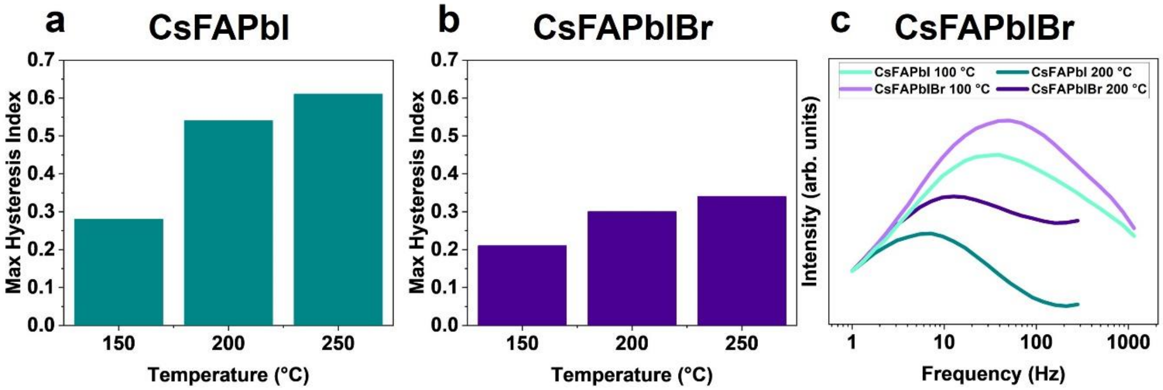

Figure S15. Maximum Hysteresis and IMPS with Changing Annealing Temperature. Maximum hysteresis index calculated from J-V curves from full solar cells for a) CsFAPbI and b) CsFAPbIBr. C) IMPS in the imaginary part of the current response in the low frequency range with background correction.

Hysteresis Index

The hysteresis index is calculated using the difference between the power measured during forward and reverse J-V curves divided by the reverse. No hysteresis will have a value of 0, while maximum will have a value of 1. Hysteresis increases with increasing annealing temperature, as is expected due to the increased production of defects. However, hysteresis is a complex process that is affected by many factors other than defects, including morphology, grain boundaries, and inherent energetics. Thus, the results here cannot be attributed solely to changing vacancies with changing composition, as adding bromine also effects properties such as morphology as seen by CL-SEM (Figure 2).

IMPS Analysis

Low frequency IMPS measurements can be used to assess ionic movement by evaluating the changing peak position. A shift to lower frequencies indicates slower ionic transport. This is expected at higher annealing temperatures due to the likely production of defects (hypothesized to be halide vacancies). This decrease in speed of ionic transport at higher annealing temperatures supports the production of vacancies hypothesized by DFT and XPS.
